# miR-623 suppresses cell proliferation, migration and invasion through direct inhibition of XRCC5 in breast cancer

**DOI:** 10.18632/aging.103182

**Published:** 2020-06-05

**Authors:** Qing Li, Jiangtao Liu, Yanli Jia, Tingting Li, Mei Zhang

**Affiliations:** 1 Department of General Surgery, Shandong Provincial Qianfoshan Hospital, The First Affiliated Hospital of Shandong First Medical University, Jinan 250000, Shandong, P.R.China; 2 Department of Internal Medical Oncology, Binzhou Central Hospital, Binzhou 251700, Shandong, China; 3 Anesthesia department, Binzhou Central Hospital, Binzhou 251700, Shandong, China

**Keywords:** microRNA, breast cancer, XRCC5, proliferation, migration

## Abstract

Background/Aims: MicroRNAs (miRNAs) are short, non-coding RNA molecules that control gene expression trough negative translational regulation. MiR-623 is a tumor suppressor, and it’s function and mechanism in breast cancer has not been reported.

Results: Exogenous overexpression of miR-623 suppressed cell proliferation, migration and invasion, meanwhile, but promoted cell apoptosis. MiR-623 knockdown displayed opposite results. Overexpression of miR-623 resulted in the downregulation of CDK4/6 as well as the inhibition of the phosphatidylinositol-3-kinase (PI3K)/Akt and Wnt/β-Catenin signaling pathways. MiR-623 knockdown displayed opposite results. Results of the reporter assay revealed that the luciferase activity was decreased in XRCC5-wt cells, suggesting that miR-623 could directly combine with 3’ UTR of XRCC5. MiR-623 significantly suppressed XRCC5 expression, which is critical for miR-623-induced proliferation and migration block in breast cancer cells.

Conclusion: miR-623 suppressed cell proliferation, migration and invasion through downregulation of cyclin dependent kinases and inhibition of the phosphatidylinositol-3-kinase (PI3K)/Akt and Wnt/β-Catenin pathways by targeting XRCC5.

Methods: miR-623 was either overexpressed in breast cancer cell lines through exogenous transfection or knocked down by specific siRNA. Cell proliferation, migration and invasion were examined using CCK-8, colony formation and transwell assay. The direct target of miR-623 was verified using luciferase reporter gene assay.

## INTRODUCTION

MicroRNAs (miRNAs) are a class of small highly conserved non-coding RNA molecules consisting of 21-25 nucleotides (nts) that are widely expressed in eukaryotic cells [1]. miRNA genes are transcribed by RNA polymerase II (pol II), generating hairpin precursors (pri-miRNAs) that undergo a series of RNA III enzyme dependent cleavage events to form mature miRNAs [2, 3]. Mature miRNAs are single-stranded RNA molecules formed, by perfect or near perfect base pairing with the target mRNA 3′-untranslated regions (3′-UTRs) leading to translational repression and/or mRNA cleavage [4–6]. miRNAs are involved in a wide range of biological processes including cell death, cell proliferation, cell division, differentiation and tumorigenesis [7–10].

The most important functional groups of miRNA are those that participate in human tumorigenesis. Numerous miRNA molecules have been shown to involved in various processes in tumorigenesis, including cancer cell proliferation, migration and invasion [11–13]. At present, many studies have demonstrated that miRNAs are capable of as tumor suppressors in various types of human cancers. MiR-623 is a tumor suppressor. A recent study shows that miR-623 inhibited the metastasis through directly suppressing MMP1 in pancreatic cancer [14]. Additionally, another study discovered that miR-623 suppressed the proliferation via targeting Cyclin D1 in gastric cancer [15]. Despite the significant progresses that has been made in different studies, the role of miR-623 in human breast cancer tumorigenesis remains unexplored. Therefore, we chose to investigate the role of miR-623 in this paper. To the best of our knowledge, this is the first study to investigate the mechanism of miR-623 in breast cancer progression.

We began our experiment by overexpressing miR-623 in breast cancer cell lines MDA-MB-453 and MCF7. Based on these cell lines, we found exogenous overexpression of miR-623 suppressed cell proliferation, migration and invasion; however, this promoted cell apoptosis. In contrary, knockdown of miR-623 demonstrated opposite results. At the molecular level, further research has revealed that miR-623 resulted in downregulation of Cyclin-Dependent Kinase (CDK) 4/6 in MCF7 cells, and inhibition of the phosphatidylinositol-3-kinase (PI3K)/Akt/mTOR and Wnt/β-Catenin signaling pathways in MDA-MB-453 and MCF7. Moreover, we proved that miR-623 directly targets XRCC5, which is critical for miR-623-induced proliferation and migration blockage in breast cancer cells.

## RESULTS

### MiR-623 is down-regulated in human breast cancer cells

**Figure 1:**
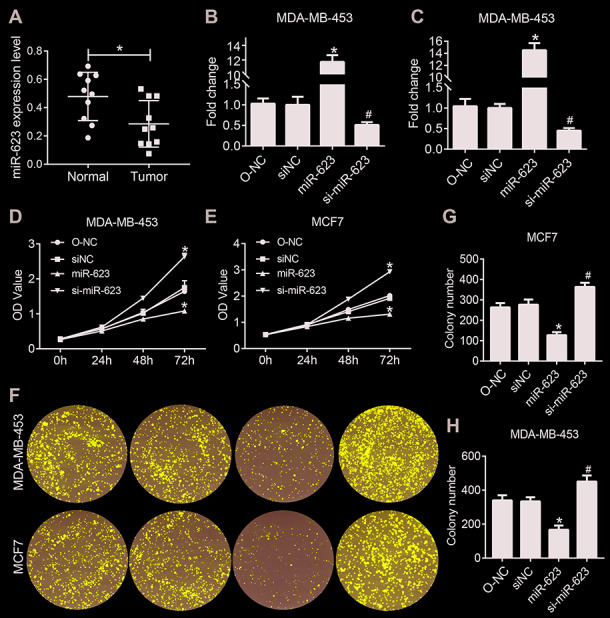
**miR-623 inhibited cell growth.** (**A**) Expression levels of miR-623 were detected in human normal mammary cell line MCF-10A and breast cancer cell lines BT474, MDA-MB-231, MDA-MB-453 and MCF-7 by real-time PCR. MiR-623 expression in MDA-MB-453 cells (**B**) and MCF7 cells (**C**) was determined by real-time PCR after transfection. Cell proliferation of MDA-MB-453 (**D**) and MCF7 (**E**) after the suppression or overexpression of miR-623 was evaluated by CCK-8 assay. (**F**–**H**) Colony formation assay of MDA-MB-453 and MCF7 cells showed that miR-623 knockdown inhibited the proliferation, whereas miR-623overexpression promoted the proliferation in breast cancer cell lines. One of the three repeated experiments with identical results was shown. *P* values were determined using Student’s t-tests. *P<0.05 compared with O-NC. #*P*<0.05 compared with siNC.

The expression of miR-623 in human normal mammary cell line MCF-10A and breast cancer cell lines BT474, MDA-MB-231, MDA-MB-453 and MCF-7 was determined by quantitative real-time RT-PCR (qRT-PCR). As shown in Figure 1A, the miR-623 level in human normal mammary cell line was significantly higher than that in breast cancer cell lines (MDA-MB-231, MDA-MB-453 and MCF-7) (*P*<0.05). The abnormal expression of miR-623 indicates that it may play a role in the origin and development of breast cancer.

### MiR-623 is transfected successfully in breast cancer cell lines

In order to evaluate the role of miR-623 in the two selected human breast cancer cell lines, it was transfected into MDA-MB-453 and MCF7 cells, and qRT-PCR was used to quantify its expression. As shown in Figure 1B and 1C, we found that the expression of miR-623 was significantly increased in the miR-623 group and decreased in the si-miR-623 group cells (P < 0.05). The overexpression and knockdown of miR-623 was confirmed before further analysis.

### MiR-623 inhibits breast cancer cell proliferation

To determine the effect of miR-623 on cell proliferation, we performed CCK-8 assay with MDA-MB-453 and MCF7 cells. The CCK-8 assay showed that cell proliferation capacity was significantly decreased in miR-623 ovexpressed cells and increased in miR-623 knockdown cells (P < 0.05, Figure 1D and E). To further confirm the inhibitory effect of miR-623 on cell proliferation, we also carried out a colony formation assay. As shown in Figure 1F–1H, overexpression of miR-623 in MDA-MB-453 and MCF7 cells induced much fewer and smaller colonies compared with the control cells (*P* < 0.05). In contrary, miR-623 knockdown resulted in opposite results. These data indicated that miR-623 dramatically suppressed breast cancer cell proliferation.

### MiR-623 attenuates the expression of CDK4 and CDK6

**Figure 2:**
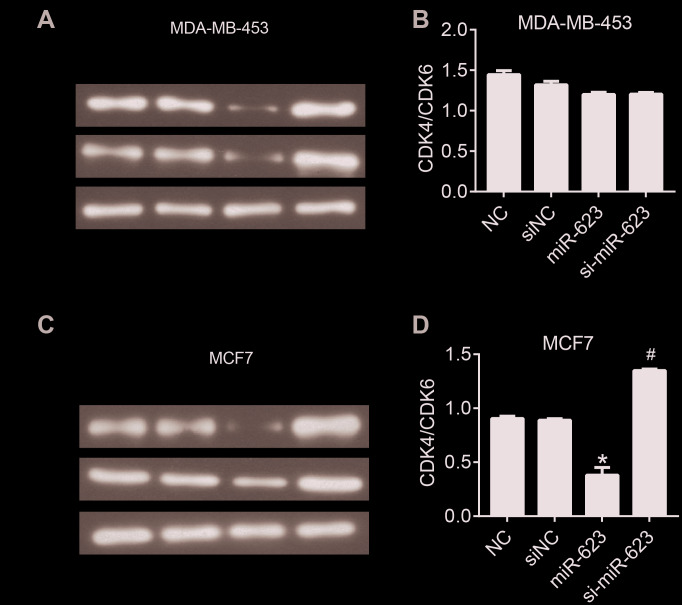
**miR-623 inhibited the expression of cell cycle proteins.** The levels of CDK4 and CDK6 in MDA-MB-453 cells (**A**) and MCF7 cells (**C**) were detected using western blot assay. The level of CDK4/6 in MDA-MB-453 cells (**B**) and MCF7 cells (**D**). GAPDH was the internal control. Relative amounts of proteins normalized to GAPDH were shown. Experiments showing identical results were performed twice. *P* values were determined using Student’s t-tests. *P<0.05 compared with O-NC. #*P*<0.05 compared with siNC.

Tumor progression is usually accompanied with dysregulation of the cell cycle and subsequent uncontrolled cell proliferation. To further investigate the anticancer activities of miR-623 on the growth of MDA-MB-453 and MCF7 cells, we examined the expression of cyclin-dependent kinase (CDK4 and 6), which are known to play an important role in the cell cycle. In the present study, we performed western blot analysis to determine the expression of CDK4 and 6. As shown in Figure 2, overexpression of miR-623 vigorously decreased the level of CDK4/6 compared to the control group, and knockdown of miR-623 increased CDK4/6 levels in MCF7 cells (*P* < 0.05). However, we did not observe this trend in MDA-MB-453 cells. Elevated expression of miR-623 has been determined to inhibit cell proliferation which may be associated with an uncontrolled cell cycle. Different results in the two cell lines also suggested that there might be other pathways for the regulation of proliferation via miR-623.

### MiR-623 inhibits cell migration and invasion

**Figure 3:**
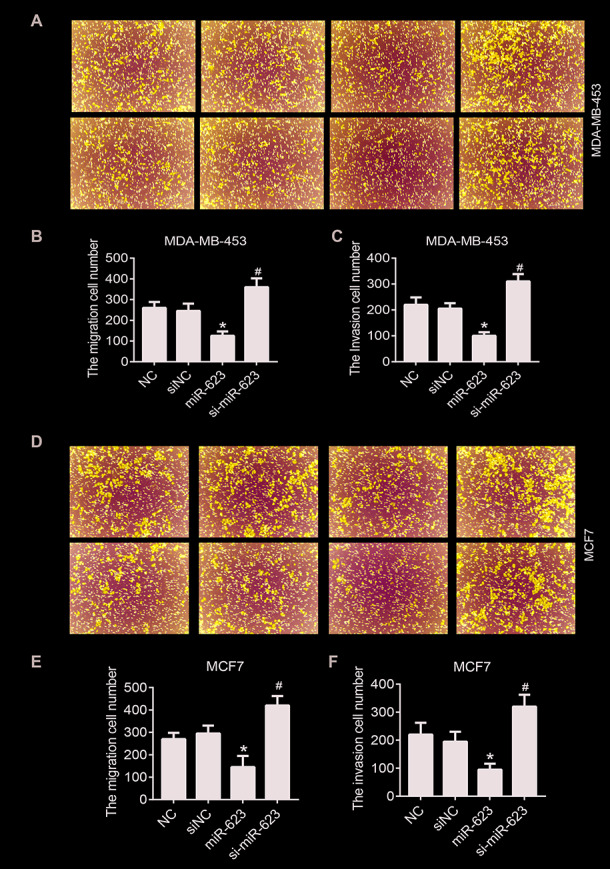
**Effects of miR-623 on cell migration and invasion.** The migration and invasion of MDA-MB-453 cells (**A**–**C**) and MCF7 cells (**D**–**F**) were analyzed by transwell migration assays and matrigel invasion assays, respectively. 10% FBS was used as the chemoattractant. Results are represented from three independent experiments. *P* values were determined using Student’s t-tests. *P<0.05 compared with O-NC. #*P*<0.05 compared with siNC.

To further investigate the effect of miR-623 in regulating cell motility, Transwell assays were used to evaluate the role of miR-623 on MDA-MB-453 and MCF7 cells migration and invasion. As shown in Figure 3A–3C, the invasive capability of cells with high level of miR-623 was inhibited significantly, while it was promoted in cells with low levels of miR-623 (*P* < 0.05). Similarly, overexpression of miR-623 resulted in a significant decrease in cell migration ability, and miR-623 knockdown resulted in opposite results (*P* < 0.05) (Figure 3D–3F). These results suggest that miR-623 is able to suppress the ability of breast cancer cells to invade and migrate.

### MiR-623 promotes cell apoptosis

**Figure 4:**
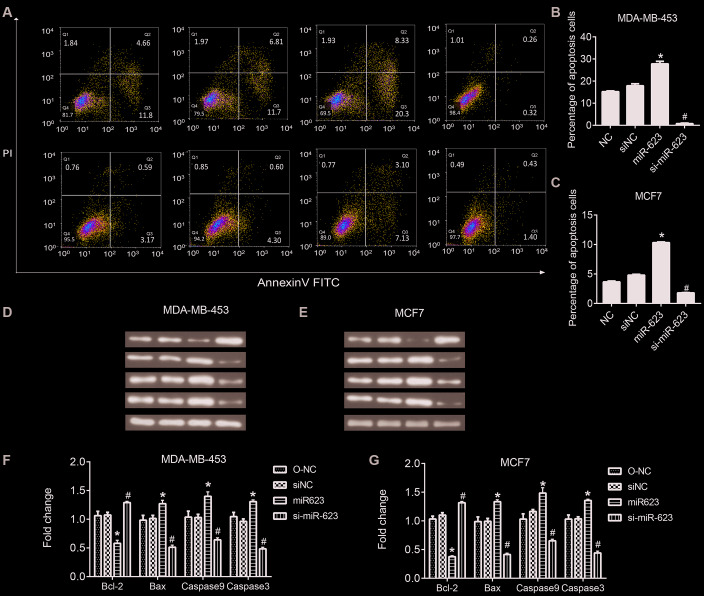
**Effects of miR-623 on cell apoptosis.** (**A**–**C**) The apoptosis of MDA-MB-453 and MCF7 cells was determined using double staining with annexin V/propidium iodide (PI) by flow cytometry. (**D**–**G**)The protein levels of apoptosis-related genes in MDA-MB-453 cells and MCF7 cells were detected by western blot assay. GAPDH was the internal control. Relative amounts of proteins normalized to GAPDH were shown. Experiments showing identical results were performed three times. *P<0.05. *P* values were determined using Student’s t-tests. *P<0.05 compared with O-NC. #*P*<0.05 compared with siNC.

It is well known that apoptosis is associated with the mechanism of tumor progression. Therefore, we next detected whether the overexpression of miR-623 would have any effects on cell apoptosis. Flow cytometry and western blot analysis were performed to evaluate apoptosis of MDA-MB-453 and MCF7 cells (Figure 4A). The flow cytometry results showed that upregulated miR-623 could promote cell apoptosis, and downregulated miR-623 could inhibit cell apoptosis compared to the NC group (*P* < 0.05, Figure 4B and 4C). These results were further validated by western blot assay. We examined the expression of Bcl2, Bax, Caspase 9 and Caspase 3 proteins. Bcl2 is an anti-apoptotic protein and Bax is a pro-apoptotic protein, while Caspase 9 is an apoptotic initiator and Caspase 3 is an apoptotic executioner. these proteins play important roles in the process of apoptosis. The western blot results showed that overexpression of miR-623 down-regulated Bcl2expression and up-regulated the expression of Bax, Caspase 9 and Caspase 3. miR-623 knockdown resulted in opposite results (P < 0.05, Figure 4D–4G). Collectively, these data suggested that miR-623 could promote breast cancer cell apoptosis.

### MiR-623 suppresses PI3K/AKT and Wnt/β-catenin signaling pathways

**Figure 5:**
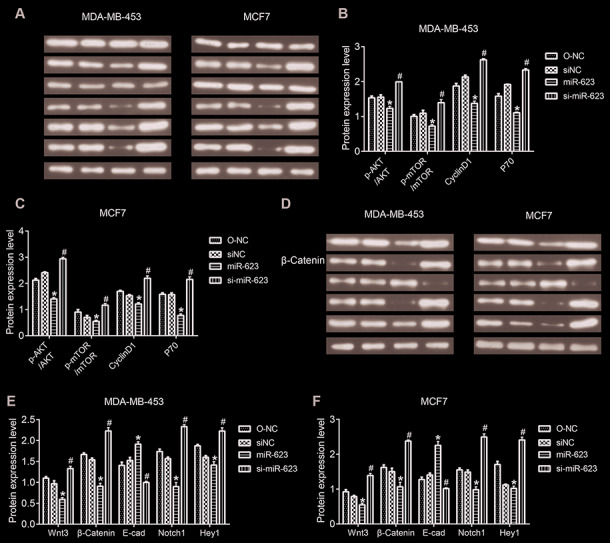
**Effects of miR-623 on PI3K/AKT and Wnt/β-catenin signaling pathways.** (**A**–**C**) Protein expressions of AKT, p-AKT, mTOR, p-mTOR, Cyclin D1, S6K/P70 in MDA-MB-453 and MCF7 cells were detected by western blot assay. (**D**–**F**) Protein expression levels of wnt3, β-catenin, E-cad, Notch1, and HEY1 in MDA-MB-453 and MCF7 cells were detected by western blot assay. GAPDH was the internal control. Relative amounts of proteins normalized to GAPDH were shown. Experiments showing identical results were performed three times. *P* values were determined using Student’s t-tests. *P<0.05 compared with O-NC. #*P*<0.05 compared with siNC.

The PI3K/AKT/mTOR and Wnt/β-catenin signaling pathway play important roles in proliferation and survival of breast cancer. Therefore, we decided to eventually investigated the involvement of both PI3K/AKT and Wnt/β-catenin signaling pathways in tumor progression. Western blot results showed that miR-623 inactivated the PI3K/AKT signaling pathway by decreasing expression of phosphorylated AKT (p-AKT), phosphorylated mTOR (p-mTOR), Cyclin D1 and S6k/P70, but without predominantly affecting total AKT and mTOR (P < 0.05, Figure 5A–5C). Likewise, we examined the Wnt/β-catenin signaling pathway related protein wnt3, β-catenin, Notch1, and HEY1. The expression levels of these proteins were also downregulated; however, E-cad expression was upregulated in the miR-623 overexpressed MDA-MB-453 and MCF7 cells. In contrary, miR-623 knockdown resulted in opposite results. (P < 0.05, Figure 5D–5F). Altogether, the suppression of these downstream effectors in the two signaling pathways demonstrated the effects of miR-623 on breast cancer cell proliferation, cell cycle and metastasis.

### MiR-623 directly targets XRCC5 in breast cancer cells

**Figure 6:**
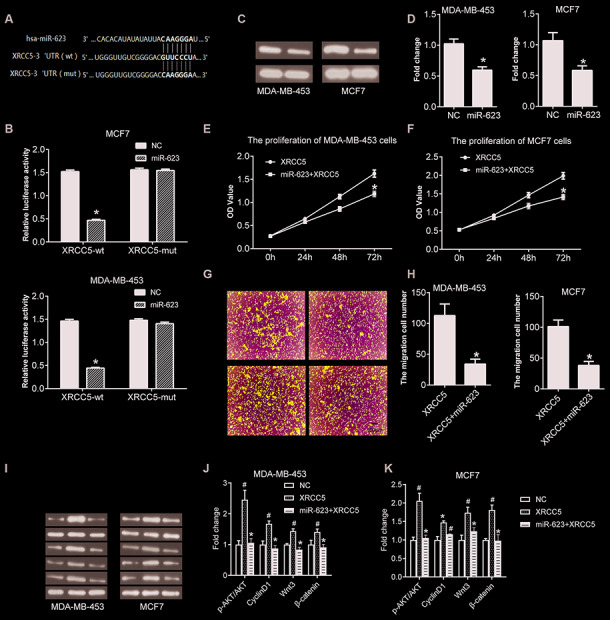
**miR-623 inhibited XRCC5 expression and blocked cell growth by targeting XRCC5.** (**A**) miR-623 and XRCC5 binding sites predicted by targetscan. (**B**) MDA-MB-453 and MCF7 cells were transfected with reporter plasmids followed by transfection with pCMV-miR-623 or pCMV-miR empty vector. The luciferase expression was analyzed as described in the “Material and Methods” section. (**C**, **D**) The XRCC5 expression was detected in NC and miR-623 cells using western blot. GAPDH was the internal control. The proliferation of (**E**) MDA-MB-453 cells and (**F**) MCF7 cells were confirmed by CCK8 assay to investigate the target of miR-623-induced cell growth block. (**G**, **H**) Transwell assay was performed to confirm the cell migration in XRCC5 and XRCC5+miR-623 cells. (**I**) The expression levels of protein in PI3K/AKT and Wnt/β-catenin signaling pathways were detected by western blot in (**J**) MDA-MB-453 cells and (**K**) MCF7 cells *P < 0.05 *vs.* XRCC5; # *P* < 0.05 *vs.* NC. *P* values were determined using Student’s t-tests.

In general, miRNAs modulate target mRNA expression through binding to the 3' untranslated region (UTR), which in turn affects cells biological behaviors. Therefore, the analysis and identification of miR-623’s target genes has great significance for the study of miR-623 function. First, miR-623 target prediction was performed by Targetscan online software. The results showed that there is a binding site for the miR623 and XRCC5 3’ UTR regions, the specific pairing information of which was shown in Figure 6A. Subsequently, the binding of miR623 to XRCC5 in the cell was verified using a luciferase reporter assay. The constructions of the reporter vector and reporter gene assay were performed as described in the material method section. As shown in Figure 6B.

The luciferase activity was decreased in XRCC5-wt group cells. This was not impacted in the XRCC1-mut cells, suggesting that miR-623 could directly combine with 3’ UTR of XRCC5. Next, we evaluated whether miR-623 had an effect on the expression of XRCC5 in breast cancer cells using western blot analysis. Our results indicate that overexpression of miR-623 significantly inhibited XRCC5 expression both in MDA-MB-453 and MCF7 cells (Figure 6C and 6D).

### XRCC5 is critical for miR-623-induced proliferation and blockage of migration

To detect whether XRCC5 is required for miR-623-induced inhibition of cell growth, we generated some cells with both miR-623 and XRCC5 overexpression, and some cells with only XRCC5 overexpression. CCK8 and transwell for migration assays were performed after transfection for 48h. According to the results shown in Figure 6E and 6F, overexpression of miR-623 and XRCC5 inhibited cell proliferation compared with XRCC5. The results demonstrate a consistent decrease in migration capacity of MDA-MB-453 (Figure 6G) and MCF7 (Figure 6H) cells with overexpression of miR-623 and XRCC5.

### MiR-623 blocked the activations of PI3K/AKT and Wnt/β-catenin signaling pathways induced by XRCC5

Then, we detected the PI3K/AKT and Wnt/β-catenin signaling pathways in XRCC5 overexpressed cells. As shown in Figure 6I–6K, the transfection of XRCC1 overexpression plasmid increased the level of p-AKT/AKT and Cyclin D1, as well as the level of Wnt3 and β-catenin both in MDA-MB-453 and MCF7 cells. However, the overexpression of both miR-623 and XRCC5 significantly inhibited the increase of level of p-AKT/AKT, Cyclin D1, Wnt3 and β-catenin induced by XRCC5 overexpression (Figure 6I–6K). These data indicated that miR-623 inhibited the PI3K/AKT and Wnt/β-catenin signaling pathways through targeting XRCC5 in breast cancer cell.

## DISCUSSION

Previous studies have shown that miR-623 acts as a tumor suppressor in pancreatic and gastric cancer [14, 15]. However, we believe a variety of research is needed to elucidate the biological importance of miR-623. Thus, we examined the effect of miR-623 expression on breast cancer.

Breast cancer is the most frequently diagnosed cancer and the second leading cause of cancer death in female worldwide [16–18]. In this study, we observed that miR-623 suppressed cell proliferation of breast cancer cells (MDA-MB-453 and MCF7); cell proliferation is a complicated process involving many different factors. Then, we found that miR-623 upregulated CDK4/6 protein level in MCF7 cells. It is well known that CDK4 and its functional homologue CDK6 play key roles in mammalian cell proliferation, where they help drive the progression of cells into the DNA synthesis (S) phase of the cell-division cycle. Additionally, the cyclin D1–CDK4/6 axis plays a central role in breast cancer development and maintenance [19–22]. Therefore, we conclude that the suppressive effects of CDK4 and CDK6 expression by miR-623 not only affect cancer cell proliferation, but also influence cancer cell migration and invasion.

Migration and invasion of cancer cells account for the metastasis and deterioration of breast cancer. Through *in vitro* cell migration and invasion assay (transwell), we found that miR-623 was able to significantly inhibit migration and invasion in both MDA-MB-453 and MCF7 breast cancer cells. Moreover, our experimental results show that miR-623 promoted breast cancer cell apoptosis. Apoptosis which is also known as programmed cell death, plays a crucial role in developing and maintaining the health of the body by eliminating old and unhealthy cells [23]. It is well known that inhibition of apoptosis plays a critical role in cancer cell survival and tumor development [24]. Therefore, the results of this study areconsistent with already established information.

Meanwhile, we discovered that the miR-623 suppressed both PI3K/AKT/mTOR and Wnt/β-catenin signaling pathways. Whether PI3K/AKT/mTOR pathway or Wnt/β-catenin pathway is dysregulated in human cancer development? The PI3K/AKT/mTOR signaling pathway plays an important role in proliferation and survival of breast cancer cells [25, 26]. Therefore, the inhibition of this pathway by miR-623 seems to be a promising therapeutic target in breast cancer treatment [25, 27]. The Wnt/β-catenin signaling pathway regulates the self-renewal and migration of cancer stem cells (CSCs), thereby promoting tumor growth and metastasis in breast cancer [28, 29]. It is important to note that miR-623 promoted the upregulation of E-cadherin protein. E-cadherin is expressed in epithelial cells, and its expression is decreased during epithelial-mesenchymal transitions (EMT) [30, 31]. EMT is a process by which epithelial cells morphologically transform into cells with a mesenchymal phenotype [32, 33], and plays a very important role in breast cancer metastasis [34]. According to our results, miR-623 plays a role in this process by suppressing EMT, thereby preventing breast cancer metastasis.

The human XRCC5 gene is located on chromosome 2q33-35 [35]. The Ku80 and Ku70 proteins encoded by XRCC5 and XRCC6, respectively, form a heterodimer (Ku protein), which is a conserved DNA adhesion protein involved in the repair of DNA double strand breaks and plays an important role in maintaining the functional integrity of the genome and repairing the damage caused by carcinogenic factors [36, 37]. Previous studies have shown that abnormal expression of XRCC5 is associated with increased risk of cancer [38–40]. In the present study, we indicated that miR-623 could inhibit the expression of XRCC5 and thus affect cell function. Our results are consistent with previous research. It has been proven that hsa-miR-526b suppresses cell growth by downregulating Ku80 in non-small cell lung cancer [41]. However, the specific mechanism by which miR623 interferes with cell growth through XRCC5 is yet to be elucidated. In addition, the direct downstream target and regulatory mechanism of XRCC5 on the biological behavior of breast cancer cells remains unknown. We predict that there are still unknown mechanisms for the action of XRCC5. We will continue to explore it in future research.

In conclusion, this study uncovers the novel functions of miR-623 in breast cancer cells. We determined that miR-623 suppressed breast cancer cell proliferation, migration, and invasion but promoted apoptosis. Meanwhile, this was accompanied with the inhibition of the PI3K/AKT/mTOR and Wnt/β-Catenin signaling pathways. Moreover, the above function of miR-623 may be achieved by suppressing the expression of XRCC5. The most important finding from this experiment is that miR-623 is a new potential marker for breast cancer diagnosis and therapy.

## MATERIALS AND METHODS

### Cell culture and transfection

The human breast cancer cell lines (MDA-MB-453 and MCF7) were purchased from Cell Bank of the Chinese Academy of Sciences (Shanghai, China). MDA-MB-453 cells were cultured in Dulbecco’s Modified Eagle Medium with 10% fetal bovine serum (Hyclone), supplemented with 100 U/mL penicillin and 0.1 mg/mL streptomycin. MCF7 cells were cultured in RPMI 1640 medium (Hyclone) containing 10% fetal bovine serum, 100 U/mL penicillin and 0.1 mg/mL streptomycin. These two cell lines were cultured in a 5% CO_2_ incubator at 37 °C. Cells were seeded into 6-well plates while in their exponential growth phase. The 80% confluent cells were transfected with one of the following: pCMV-miR-623 plasmid (miR-623 group), pCMV-miR empty vector (O-NC group), miR-623-specific-siRNA (si-miR-623 group), negative control siRNA (siNC group), pcDNA3.1-XRCC5 plasmid (XRCC5 group) or XRCC5-specific-siRNA (siXRCC5 group), reporter plasmid with Lipofectamine™ 2000 (Life Technologies) according to manufacturer’s protocol for 24 hours for the further experiments.

### RNA extraction and quantitative real-time RT-PCR assay

Total RNA was extracted from transfected MDA-MB-453 and MCF7 cells using TRIzol (Invitrogen). Then 1 μg of RNA was reverse transcribed to cDNA using miRNA First-Strand cDNA Synthesis Kit (Invitrogen) according to the manufacturer’s instruction. miR-623 expression was assessed using quantitative real-time RT-PCR (qRT-PCR) with the SYBR Green PCR Master Mix (Takara) according to the manufacturer’s protocol. The qRT-PCR reactions were performed as follows: 95 °C for 5 min, followed by 40 cycles of 95 °C for 15 sec, 60 °C for 45 sec, and finally maintained at 72 °C for 30 min. U6 was used to normalize expression. All reactions were performed in triplicate. Data analysis was performed according to the 2^-ΔΔ^Ct method.

### Cell viability and colony formation assays

After transfection for 24 hours, the transfected MDA-MB-453 and MCF7 cells were seeded into 96-well plates (1×10^3^ cells per well). Cell proliferation was measured at 24, 48 and 72 hours by cell counting kit-8 assay (Promega Corporation). The absorbance of each well was detected at 450 nm using a spectrophotometer (Thermo Fisher Scientific). For the colony formation assay, the transfected cells were seeded and maintained in 6-well plates (2×10^3^ cells per well) for 2 weeks. Then colonies were fixed with 4% paraformaldehyde for 15 min and stained with Giemsa for another 15 min. Finally, the colonies were visualized under an inverted microscope and counted in five random fields for further statistical analysis.

### Cell migration and invasion assay

For the cell invasion assay, 100 μL transfected MDA-MB-453 and MCF7 cells (1×10^5^ cells per well) in serum-free medium were seeded into the Matrigel-coated upper chamber of transwell inserts (Corning), whereas 500 μL medium was added into the lower chamber. After incubation for 12 hours at 37 °C, the non-invasion cells on the upper chamber were removed using cotton swabs, then the cells that invaded through the Matrigel-coated filters were fixed with 4% paraformaldehyde for 15 min and stained with 0.1% crystal violet. The number of cells on the lower surface of the filter was counted using a microscope in five random fields, and all samples were performed in triplicate. The migration assay was the same as the invasion assay except that the top chambers were not coated with Matrigel (BD Biosciences).

### Western blot assay

48 hours later after transfection, the cells were washed once in cold PBS then lysed in RIPA buffer with protease and phosphatase inhibitor cocktails (Sigma) for 10 min. Cell lysate was centrifuged at 12000 rpm for 10 min at 4 °C and the supernatants were collected. Protein concentration was determined by BCA protein assay. 20 μg total protein samples were analyzed on SDS-PAGE and transferred onto the PVDF membrane (Millipore). The PVDF membrane was blocked with TBST (100 mM Tris-HCl pH 7.5, 150 mM NaCl, 0.05% Tween 20) with 5% non-fat dried milk for 1 h, then incubated with primary antibodies overnight at 4°C. The next day, the HRP-coupled secondary antibody was added and protein bands were visualized using ECL reagents (Pierce). GAPDH was used as a protein-loading control.

### Apoptosis detection by flow cytometry

Cells were transfected with plasmid using Lipofectamine™ 2000 (Life Technologies) according to the manufacturer's instructions. Twelve hours after transfection, the culture medium was replaced with DMEM without FBS and cells were starved for an additional 24 hours. Cells were collected and washed once with PBS, then adjusted to 5×10^6^ cells/ml. 5 μL Annexin V/FITC and then 10 μL PI were incubated with 100 μL cell suspension for 10 minutes before FACS analyses. The results were analyzed by Flowjo software.

### Luciferase reporter gene assay

The XRCC5 wildtype and mutant reporter plasmids were constructed for luciferase reporter gene assay. A XRCC5 3’ UTR fragment containing the binding site predicted by targetscan was recombined into a pGL3.0 vector to generate the XRCC5-wt plasmid; and a mutated XRCC5 3’ UTR fragment was recombined into the luciferase construct to generate the XRCC5-mutant plasmid. All plasmids were verified by sequencing. Cells were transfected with pCMV-miR-623 and reporter plasmids as miR-623 overexpression group, and transfected with pCMV-miR empty vector and reporter plasmids as control group. Forty-eight hours following the transfection, the cells were lysed in lysis buffer. The luciferase expression was determined by Dual-Glo Luciferase Assay System (Promega, USA).

### Statistical analysis

Data from individual experiments were presented as Mean±SD. Statistical significances were determined using the unpaired *T*-test. *P*<0.05 was considered to be statistically significant.
